# Supplementary material for: Ballistic transport and boundary resistances in inhomogeneous quantum spin chains
Source: Nat Commun. 2019 Oct 23;10:4820. doi: 10.1038/s41467-019-12784-4 (PMC6811644; doi:10.1038/s41467-019-12784-4)
Supplement: Supplementary file 1 — Supplementary Information [file 41467_2019_12784_MOESM1_ESM.pdf]

Supplementary Information to:

Ballistic transport and boundary resistances in inhomogeneous quantum spin chains

Biella *et al.*

Supplementary Note 1: Thermalization dynamics for spin transport — In this section we show the thermalization dynamics for the spin transport scenario. In analogy to the analysis done in the main text for the energy transport scenario, we study the dynamics of the local effective chemical potential $\mu_n^{\text{eff}}(t)$ (see Methods for details). In the panel (a) of Supplementary Fig. 1 we show the effective chemical potential profiles at different times for a fixed anisotropy. The data show the progressive emergence of a discontinuity at the junction. Again, as in the case of energy transport, this behavior can be understood in terms of a magnetic Kapitza boundary resistence. In panel (b) of Supplementary Fig. 1 we show the profile of μ_n^{eff} for the longest time accessible with our numerics (i.e. $t = 40$) as a function of the anisotropy parameter γ. While in the homogenous case $\gamma = \pi/2$ the profile is smooth, for $\gamma \neq \pi/2$ the discontinuity increases as the anisotropy is increased.

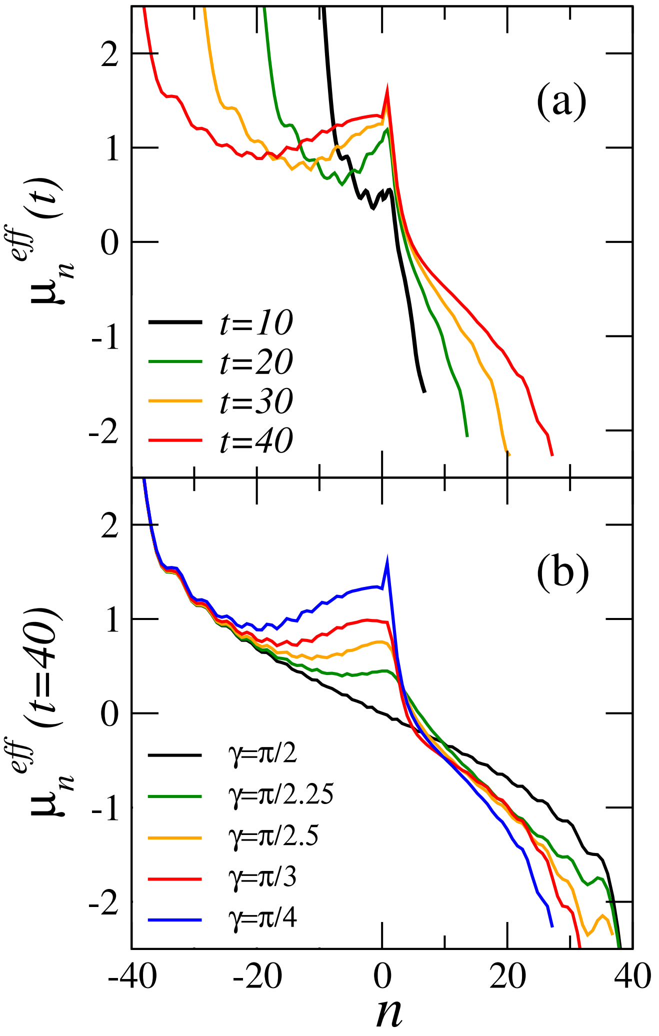

Supplementary Fig. 1. **Chemical potential profiles.** Effective chemical potential: panel (a), profile of μ_n^{eff} for $\gamma = \pi/4$ and several values of time; panel (b), profile of μ_n^{eff} at the longest accessible time, $t = 40$, and several values of γ.

Supplementary Note 2: Absence of thermalizing tendency in a homogeneous integrable model — In this section we show the the absence of thermalizing tendency when $\gamma = \pi/2$ (homogeneous case). In analogy with the analysis performed in the main text for inhomogeneous systems, we study the distance between the local reduced three-site density matrix $\rho_{n,n+2}(t)$ and the thermal density matrix $\rho_{n,n+2}[\beta_n^{\text{eff}}(t)] = e^{-\beta_n^{\text{eff}}(t)\hat{H}}/\mathcal{Z}$. In particular, we compute the dynamics of the trace norm $d_n\{\rho_{n,n+2}(t), \rho_{n,n+2}[\beta_n^{\text{eff}}(t)]\}$ as function of the rescaled coordinate n/t. Supplementary Fig. 2 clearly shows that homogenous systems do not display any tendency towards thermalization at the junction ($n/t = 0$). The rescaled profiles of $d_n(t)$ at different times collapse for $t \gtrsim 10$. This also shows the absence of slow relaxation regions in homogenous systems which relax to a non-equilibrium stationary state on a faster timescale.

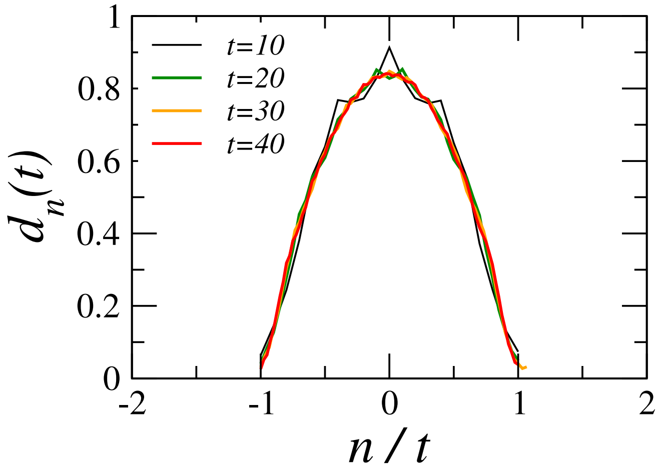

Supplementary Fig. 2. **Absence of a tendency towards thermalization.** distance between the three-site reduced density matrix $\rho_{n,n+2}(t)$ and $\rho_{n,n+2}[\beta_n^{\text{eff}}(t)]$ for several times and $\gamma = \pi/2$.
